# Uses of Antibiotics Alone in Case of Uncomplicated Appendicitis

**DOI:** 10.7759/cureus.28488

**Published:** 2022-08-27

**Authors:** Siddhartha Yadao, Yashwant Lamture, Shreyash Huse

**Affiliations:** 1 Medicine, Datta Meghe Institute of Medical Sciences, Wardha, IND; 2 Surgery, Datta Meghe Institute of Medical Sciences, Wardha, IND

**Keywords:** non operative management, appendicitis, appendectomy, broad-spectrum antibiotics, uncomplicated

## Abstract

The frequent abdominal surgical emergency is acute appendicitis with a significantly less lifelong risk. One of the most common surgeries manifested is an appendectomy, but with recent advances, non-operative management has evolved using antibiotics. In adult patients with simple appendicitis, we identified the role of surgical and non-surgical therapy. One of the most common surgeries manifested is an appendectomy, but with recent advances, non-operative management has evolved using antibiotics. In adults suffering from mild appendicitis, we identified the role of surgical and non-surgical therapy. The analysis indicated that the Antibiotics versus Primary Appendectomy in Children (APAC) did not establish non-inferiority of antibiotics vs. appendectomy with a pre-specified small margin.

In contrast to the majority of appendectomies that are carried out laparoscopically, the surgeries were almost usually open. Appendectomies, both laparoscopic and open, are not the same procedure. Antibiotic therapy is effective in about 60% of cases of simple appendicitis. A surgery-only strategy would reduce antibiotic exposure, a factor to consider in these days of antimicrobial stewardship. Therefore, studies are being conducted on whether to shift alone on antibiotics or with appendectomy to have better results with fewer complications. Future studies should focus on appendicitis features and long-term unfavorable consequences, including antibiotic resistance or Clostridium difficile colitis, most responsive to antibiotics by utilizing laparoscopic procedures as controls. Using it along with appendectomy may change the outcome showing a better prognosis.

## Introduction and background

For nearly a century, appendectomy has been the primary treatment for acute appendicitis. In the United States, around 300,000 appendectomies are performed each year. Even though appendectomy is tolerated, it is a significant surgery that may be associated with postoperative morbidity. Endoscopic retrograde appendicitis therapy is a new endoscopic treatment target for acute uncomplicated appendicitis that has received support from many sources. However, no systematic investigations in children have been performed to date, and prospective comparison data are inadequate. Furthermore, because we were concerned about the risk of future cancer in children from radiation exposure, we chose contrast-enhanced ultrasonography rather than endoscopic retrograde appendiceal radiography [1].

It is widely believed that acute appendicitis always results in perforation [2]. This belief has persisted since Fitz's initial explanation of the connection between McBurney's report on reduced death from pelvic infections following an appendectomy, and the appendix that an urgent appendectomy is required upon appendicitis diagnosis stems from this line of reasoning. Fitz and McCurney were published 40 years before antibiotics were widely accessible [3-5]. Minimal invasive treatment is effective and safe, as demonstrated in several randomized clinical trials (RCTs), with a success rate ranging from 63-85%. Such trials have been the subject of meta-analyses and thorough reviews, with positive results [6]. However, because children have unique anatomical and pathophysiologic characteristics, the clinical picture of acute appendicitis in pediatric patients differs from that in adults, making treatment choices for children more challenging. The findings from past meta-analyses and systematic reviews of adult patients do not translate to pediatric patients. Results from sizable research with pediatric patients determine the precise clinical recommendations regarding whether non-operative therapy or appendectomy should be performed for pediatric patients with acute appendicitis. Recent research suggests that in cases of pediatric appendicitis that are not complicated, antibiotic treatment may be a viable alternative to appendectomy. The research outcomes were contradictory, and the sample sizes were modest. The current meta-analysis observed the efficacy and safety of antibiotic therapy versus appendectomy in pediatric patients with uncomplicated severe stomach discomfort [7].

## Review

Antibiotics vs. appendectomy

The most frequent emergency requiring abdominal surgery in the United States is acute appendicitis [8]. It is not unexpected that a large amount of healthcare spending goes toward treating this disease, given the lifetime risk of 7-8%. It is possible to categorize acute appendicitis as complicated or uncomplicated. An abscess, phlegmon, or perforation are consequences of complicated appendicitis. The therapeutic paradigm for acute appendicitis has changed during the past ten years, mainly due to the disease's higher operational morbidity [9,10]. Today, many cases are treated without surgery with antibiotics. This method has produced satisfactory treatment, recurrence, and morbidity rates similar to how acute colonic diverticulitis is treated. However, it is important to note that, rather than the obvious advantage of antibiotic therapy, diverticulitis treatment without surgery aims to prevent the need for a colostomy and a subsequent laparotomy. Management of uncomplicated appendicitis may also need re-evaluation as complicated appendicitis treatment has developed into a less intrusive method. Uncomplicated appendicitis is treated surgically with antibiotics, whereas complicated appendicitis is treated often with surgery. Indeed, operational management has been regarded as the industry standard treatment for more than 125 years.

The American pathologist Reginald Fitz published autopsy data on the subject in 1886 when this method first appeared. He stated that the majority of appendices. Ultimately, infections lead to gangrene, perforation, and bladder abscess. These abscesses in the pelvis were fatal at that time, primarily since the study was published more than 40 years before Alexander Fleming discovered penicillin. Charles McBurney, a surgeon, died three years after Fitz. Charles McBurney, a surgeon, presented his ground-breaking research three years after Fitz, demonstrating how an early appendectomy avoids pelvic abscess. Because of his compelling findings, appendectomy has been the go-to procedure for treating acute appendicitis for over a century. Despite having almost universal support in the surgical community, According to several examinations, an appendicitis diagnosis may not always call for surgical intervention. In 1959, Coldrey published a report on his five-year experience treating more than 400 patients with appendicitis without surgery [11]. 

Non-operative management of uncomplicated appendicitis

The first case report of sailors and military personnel using antibiotics alone to treat appendicitis was published. They discovered and analyzed six randomized controlled trials. Researchers discovered that nontreatment of uncomplicated appendicitis in adults used to have a 91% likelihood of success immediately with appearance and a 71% chance after one year. Non-operative care was not related to increased severe appendicitis or major surgery in general [12]. Early retrospective investigations suggested that children with uncomplicated appendicitis can be successfully treated with antibiotics alone. A review suggested that 83% of cases showed successful antibiotic treatment in non-operative conditions [13]. It was determined that there is most likely to be successfully treated simple appendicitis with only antibiotics, increased blood pressure without elevated C-reactive protein bilirubin or, according to imaging, an appendicolith [14]. Infants with appendicitis who already have experienced stomach pain for less than a year were more than 24 hours with localized stomach pain and received non-operative treatment and hemodynamic stabilization. In their trial, non-operative treatment was effective in 15 of 16 patients, or 93.7 percent [15].

The safety and effectiveness of non-operative treatment for children with uncomplicated appendicitis have been well understood in recent clinical trials. In a study, 22 of 24 patients (92%) who received antibiotic treatment experienced a temporary improvement in their symptoms [16]. During the one-year follow-up period, only one of these 22 patients (or 5%) experienced a recurrence of acute appendicitis. Recent clinical trials have revealed more information about the effectiveness and safety of non-operative treatment for children with uncomplicated appendicitis. In research, antibiotic treatment resulted in the first remission of symptoms in 22 of 24 patients (92%). During the follow-up period, appendices were not removed from 62 percent of patients handled nonoperatively. All uncomplicated appendicitis patients were given the option to choose between surgical or non-surgical treatment upon admission; in research by Tanaka et al., 86 patients chose surgical intervention, while 78 went for non-operative treatment. A 98.7% first success rate for non-operative care led to hospital discharge. The length of hospital stay was the same for both divisions. Postoperative paralytic ileus occurs in 2 surgically treated patients, while 22 non-surgical care patients (28.6 percent) had recurrent appendicitis throughout a 4.3-year follow-up period. The presence of an appendicolith may decrease the likelihood that appendicitis will be resolved with antibiotics alone because it may obstruct the appendix's lumen, which may take longer to clear up with antibiotics alone. A recent investigation validated the risk factor for non-operative management failure, the existence of an appendicolith [17,18].

There was a need for an alternate therapy that relied primarily on antibiotics because of the long trips and the restricted availability of surgical care [19]. To learn more about non-operative treatment for adult appendicitis, current clinical data review and meta-analysis Six RCTs were identified and integrated into their analysis. Investigators found that nontreatment had a 91 percent success rate currently of presentation and a 71 percent success rate at one year for people with simple appendicitis. Non-operative care, in general, was not associated with a rise in the incidence of severe appendicitis or postoperative complications. In this population, appendicitis was diagnosed based on the criteria. Patients between the ages of 7 and 18 who met the criteria could receive surgical and medical treatment. Unlike a randomized approach, which may result in many families refusing to allow young students to be involved, the patient selection technique allows the introduction of a large cohort group that is typical of actual practice. Patients who opted for surgical intervention underwent laparoscopic surgery. If a patient displayed indicators of clinical deterioration, such as worsening pain, fevers, nausea, or other symptoms, they were moved to operational management.102 individuals in all were enrolled; 65 patients/families elected to have their appendixes removed, while 37 patients/families opted for non-operative management. The groups' initial characteristics were comparable. At 30 days and one year, non-operative management had a success rate of 89% and 76%, respectively. Recently, more prospective investigations have been conducted [20,21]. Children aged 5 to 18 years with abdominal discomfort lasting less than two days and diagnosed with acute appendicitis were treated solely with antibiotics [22]. In this trial, two doses of IV piperacillin were administered, then an oral ampicillin/clavulanate treatment lasting seven days was given. The success rate was 71% after one year (CI 50-87 percent ). Without experiencing any symptoms again, two patients chose to have interval appendicectomies. After receiving antibiotic medication, none of the trial participants experienced complicated appendicitis. Moreover, the authors provided a cost-utility analysis. In a different single-institution trial, it was found that when antibiotics were started within the first 24 hours of symptoms appearing, non-operative therapy of acute appendicitis in children aged 4 to 15 years was successful 89 percent of the time [23].

A group of patients with acute appendicitis was imaging-proven in a multi-center prospective cohort study [24]. Two of the 25 patients received delayed appendectomy; the others ranged in age from 7 to 17 years. The remaining 23 individuals did not exhibit any symptoms at an 8-week follow-up. Researchers stated that non-operatively treated patients missed fewer days of work and school and that the return of the children to routine activities was accelerated. Their findings supported the effectiveness of non-operative treatment with the caveat that patients had to be carefully selected and how comfortable they were. It is essential to consider the patients, their loved ones, and the surgeon [25].

Antibiotics

Antibiotics are the foundation of conservative treatment for acute appendicitis, including randomized control trials (RCT) and controlled clinical trials (CCT) [26]. The most popular combination is cephalosporin with nitroimidazole, followed by quinolones and penicillin with a beta-lactamase inhibitor. The WSES guideline and a global multi-center prospective observational study research are the foundation for the body of evidence. Antibiotics are often taken intramuscularly for between one and three days and then ingested for five to seven days [27]. The duration of treatment varies based on the particular course and the normalization of inflammatory indicators; the optimal treatment period is unknown [28]. The duration of treatment is unknown; it is typically defined by the diagnostic workup and the stabilization of inflammatory markers [29]. A meta-analysis of 12 randomized controlled studies and the Cochrane Review, perioperative antibiotics can lower the risk of treatment failure and the incidence of abscesses [30]. In cases of complicated appendicitis, particularly since there is an abscess, peritonitis, or free perforation, continuing antibiotics postoperatively is advised [31].

Treatment

**Figure 1:**
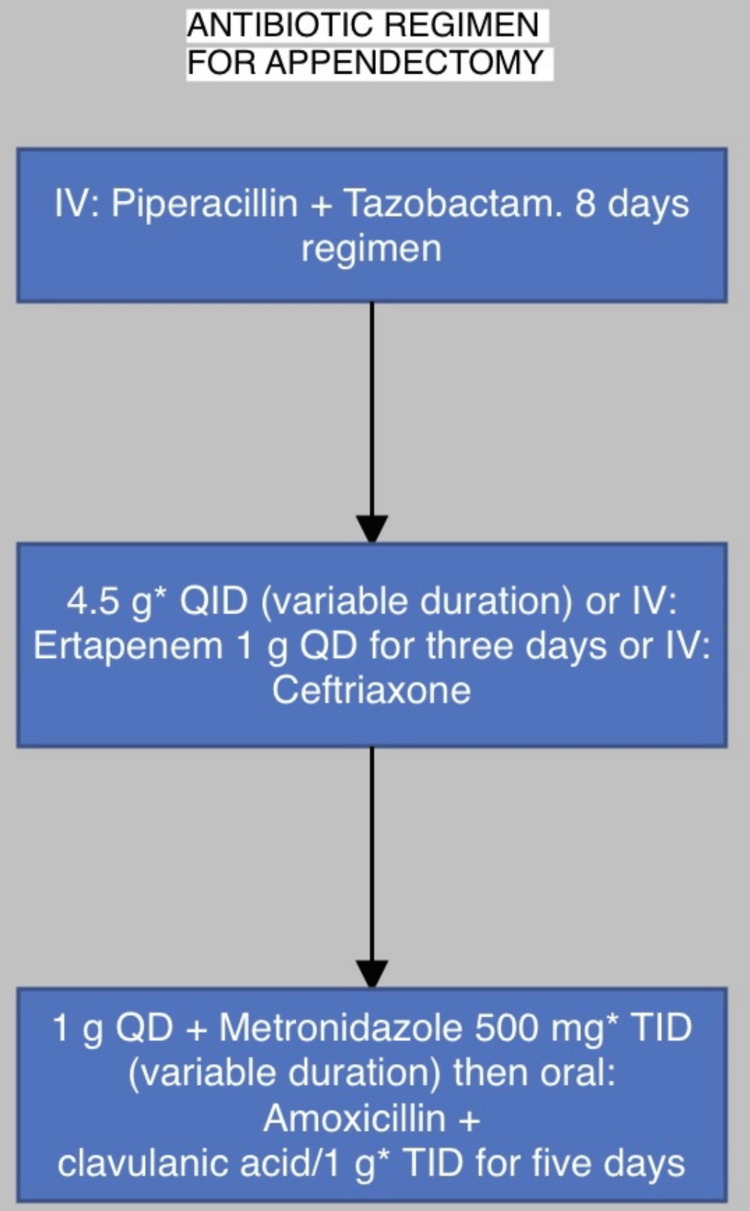
Antibiotic regimen Ref no- [34]

Appendectomies are recommended for all age groups as the therapy of choice for simple short-term appendicitis, according to the World Society of Emergency Surgery (WSES), the Society of Gastrointestinal and Endoscopic Surgeons, and the European Association for Endoscopic Surgery [32]. A few authors claimed spontaneous healing [33]. The treatment regimen for uncomplicated appendicitis is given below in figure 1.

**Figure 2:**
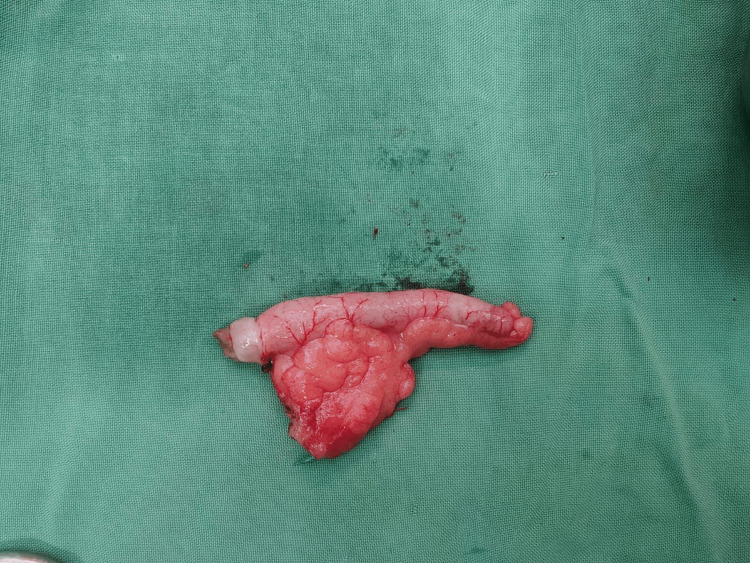
Inflamed vermiform appendix specimen (removed after appendectomy).

In 2016, a different meta-analysis revealed no complications or length of hospital stay differences. In a comparative investigation, conservative parenting in childhood resulted in higher relative risk (RR) of hospital readmission and a slower pace of remission symptoms. Due to the research examined in these papers was not conducted randomized and fewer patients than necessary were recruited being desired, it is essentially a given that the Children were more likely to receive conservative treatment. Additionally, the one-year non-inferiority criterion for antibiotic treatment was not met in the APAC trial. Although conservative treatment can be viewed as safe in all three of the evaluations mentioned above, the current level of evidence is not sufficient to support such a move. With at least one year of follow-up, According to a meta-analysis of five randomized and controlled trials published in 2019, therapeutic efficacy (i.e., lack of recurrence or appendectomy) was determined to be 62.6 percent versus 96.3 percent in the surgical group. Appendectomy was proven to be the most effective procedure for treating patients with uncomplicated appendicitis. Most antibiotic regimens were consistent with 2010 and 2017 Infectious Diseases Society of America (IDSA) and Surgical Infection Society (SIS) guidelines for the treatment of mild-to-moderate community-acquired intra-abdominal infections [35,36]. Figure 2 showing specimen of inflamed vermiform appendix removed after appendectomy.

## Conclusions

Non-operative therapy has been researched in adults and children to treat acute and complicated appendicitis. Non-operative treatment for uncomplicated appendicitis is a viable and well-tolerated approach for some patients. Non-operative therapy is another successful and well-tolerated alternative for treating acute appendicitis with a well-formed phlegmon or abscess, with or without a planned interval appendectomy. The most recent research suggests that antibiotics may be safely administered to a small proportion of patients with uncomplicated appendicitis. Clinical results should be considered with patient preferences when choosing these patients. It is contested whether using antibiotics is not worse than using the best treatment available for appendectomy. Future research should emphasize solitary employing laparoscopic procedures as comparison controls, detecting long-term adverse effects, including antibiotic resistance, and determining the features of appendicitis most amenable to antibiotics documentation of any either resistance or colitis caused by C. difficile. Taking into account the challenges of doing surgery for recurrent appendicitis and the efficacy of non-surgical antimicrobial therapy options Additionally, to improve patient choice and outcome, patient adherence to therapy must be evaluated, especially in the non-operative group.
